# Study of bronchoalveolar lavage in clinically and radiologically suspected cases of pulmonary tuberculosis

**DOI:** 10.4103/0970-2113.68307

**Published:** 2010

**Authors:** Usha Kalawat, Krishna K. Sharma, Prakash N. R. Reddy, A. Gururaj Kumar

**Affiliations:** *Department of Microbiology, Srivenkateswara Institute of Medical Sciences, Tirupati- 517 507, Andhra Pradesh, India*

**Keywords:** Bronchoalveolar lavage, fiber optic bronchoscopy, occult tuberculosis, lower respiratory tract infection, tuberculosis

## Abstract

**Context:**

About 30 to 50 % of pulmonary tuberculosis patients have sputum report negative for acid fast bacilli or present with no expectoration. A lot of research is going on to find methods to establish early and accurate diagnosis of pulmonary tuberculosis (PTB) as institutions of early treatment can have significant effects on morbidity and mortality of patients and also the development of MDR–TB. Samples other than sputum play an important role in the diagnosis of disease in such patients.

**Aims:**

To assess the significance of bronchoalveolar lavage samples and fiberoptic bronchoscopy (FOB) in the early diagnosis of occult sputum smear negative pulmonary tuberculosis.

**Settings and Design:**

Study was conducted in a tertiary care hospital. FOB was performed in patients with three consecutive sputum smear negative acid fast bacilli to obtain bronchoalveolar lavage (BAL) samples. Written informed consent was obtained from these patients.

**Materials and Methods:**

BAL samples were subjected to Z-N staining and culture on L-J slopes for acid fast bacilli. Sputum samples from the same patients were also cultured.

**Results:**

BAL samples were positive in 82.2% of sputum smear negative samples. Culture positivity of BAL samples was 90.9% as compared to sputum culture positivity which was 26.4%. Overall diagnosis could be established in 86.6% of patients with the help of fiber optic bronchoscopy.

**Conclusions:**

BAL samples are very useful in early sputum smear negative pulmonary tuberculosis and FOB can play an important role in diagnosis of lower respiratory tract infections with minimal complications in hands of an expert.

## INTRODUCTION

Mycobacterium tuberculosis (MTB), discovered by Robert Koch in 1882, is the leading killer of adults.[1] The World Health Organization (WHO) estimated 9.2 million new cases of tuberculosis (TB) in 2006 (139 per 100 000 population), including 4.1 million new smear-positive cases (44% of the total) and 0.7 million HIV-positive cases (8% of the total) worldwide. This is an increase from 9.1 million cases in 2005, due to population growth.[2] India, China, Indonesia, South Africa and Nigeria rank first to fifth respectively in terms of absolute numbers of cases. The African region has the highest incidence rate, 363 per 100 000 population.[2]

Though large proportions of pulmonary tuberculosis patients have negative AFB sputum report or present with no expectoration, the transmission rate of smear negative TB as compared to smear positive TB is reported as 22%.[3] Approximately 50% of pulmonary TB cases are sputum smear negative for AFB.[4] Published studies suggest that more than 50% of smear negative patients would need chemotherapy if left untreated.[5–6] Use of empiric Anti Tuberculous Therapy (ATT) in patients with X-ray findings strongly suggest pulmonary tuberculosis (PTB). However, repeated sputum smear negative for AFB has several disadvantages such as failure of therapy in case of multi drug resistant tuberculosis (MDR-TB), side-effects of medications and delay in diagnosis and treatment of conditions other than TB when present.[7] Therefore, samples other than sputum play an important role in patients with occult tuberculosis or other mimicking conditions.

Fiberoptic brochoscopy (FOB) has been used to obtain various kinds of samples for diagnosis of sputum smear negative pulmonary tuberculosis. The results of these studies are conflicting and inconclusive.[8–10] The overall yield of bronchoscopy for diagnosing TB has been reported as more than 90% when cultures were included in the analysis which is said to be similar even in sputum smear negative TB.[11,12]

Several studies have compared the usefulness of different samples for arriving at an early diagnosis. This study was taken at a tertiary care hospital to evaluate the significance of bronchoalveolar lavage specimen culture and acid fast staining as compared to sputum culture and staining for the diagnosis of pulmonary tuberculosis.

## MATERIALS AND METHODS

This is a prospective study conducted over a period of one year from January 2007 – December 2007. In our institute, bronchoscopy is performed by cardiothoracic surgeon for several diagnostic or therapeutic indications with informed written consent.

Bronchoalveolar lavage (BAL) samples from such 45 patients with clinical and radiographic findings suggestive of PTB with 3 consecutive Sputum smear negative for AFB were processed for diagnosis of pulmonary tuberculosis. Samples were subjected to ZN staining. Smears were examined under oil immersion lens for the presence of AFB. About 100 fields were examined for AFB before reporting negative. Microscopy findings were compared with X-ray and CT findings.

### Processing of samples for acid fast staining

BAL samples were centrifuged at about 3000 rpm for 15-20 minutes and the supernatant was transferred into another tube and smear was prepared from the sediment. Smears were fixed and stained by Z N staining. After air-drying smears were examined under oil immersion lens.

### Culture of sputum and bronchoalveolar lavage samples

Samples were digested and decontaminated using N-acetyl-L cystine. Culture was done on LJ slants following aseptic precautions. Each sample was cultured on two LJ slants. H37Rv reference strain was used as the control and was inoculated on two L J slants. Cultures were incubated at 37°c and screened for any growth at regular intervals two times a week. Cultures were considered negative for acid fast bacteria if no growth was observed after incubation of LJ slants for a period of 10 weeks. Any growth on the slants was further confirmed by ZN staining.

## RESULTS

There was no significant difference observed among the smear positive and smear negative patients with regard to clinical presentations. The X-ray and CT findings of the two groups of patients were compared and no significant difference was observed in X-ray and CT findings among the AFB positive and AFB negative patients.

In 11 patients, diagnosis was established with histopathology and cytology reports with samples collected by FOB. Biopsy was performed in only four patients of whom only one was positive for tuberculosis and no other pathology was observed. This patient’s BAL was positive for acid fast bacilli by smear as well as culture. One biopsy report was positive for small cell carcinoma of lung and other two for reactive inflammatory changes and not suggestive of tuberculosis or any malignancy. Of the 34 patients 22 (64.70%) were positive for AFB on staining of BAL samples.

Culture was positive in 28 (82.3%) of BAL samples. Of the BAL smear, positive samples culture was positive in 20 (90.9%) samples. Sputum culture was positive in nine (26.4%) patients only. Diagnosis could be established in 39 (86.6%) of the sputum smear negative samples with the help of microbiology and pathology reports.[Table 1 and 2].

**Table 1:** Smear and culture results of sputum and BAL samples (n= 34)

Sample N= 34	Smear Pos. (%)	Smear Neg. (%)	Culture Pos. (%)	Culture Neg. (%)
Sputum	0 (0)	34 (100)	9 (26.4)	25 (73.5)
BAL	22 (64.7)	12 (35.2)	28 (82.3)	6 (17.6)

**Table 2:** Additional diagnosis made by other samples collected by fiberoptic bronchoscopy

Diagnosis	No. of cases
Carcinoma lung	1
Aspergilloma	1
Right lower lobe bronchiectasis	1
Left lower lobe bronciectasis	4
Left upper lobe abscess	1
Right lower lobe pneumonia	1
Interstitial lung disease	1
Carcinoma esophagus	1
Total	11

## DISCUSSION

Since its introduction in 1968 by Ikeda *et al*. flexible bronchofibroscope has become very useful tool in patient care and medical research. Proper selection of instrument is necessary to ensure effective and safe procedure. Ability to collect BAL provides a role for flexible bronchoscope in research. The insignificant difference in the clinical presentations, X-ray and CT findings in our study suggest that though the signs and symptoms, and radiographic findings provide important clue for pulmonary tuberculosis, they cannot confirm the diagnosis of pulmonary tuberculosis. Acid fast stain positivity and culture isolation can only provide the definitive diagnosis. Therefore, patients with radiographic and clinical findings compatible with PTB but sputum smear negative are a challenge for the physician - as to start ATT or not. It has been reported that 74% of these patients develop active tuberculosis in five years if not treated.[13] Flexible fiberoptic bronchoscopy is considered as a safe diagnostic and interventional tool, even in young or extremely premature infants.[14] Caminero *et al*, concluded that bronchoscopy should be conducted on all patients without expectoration and negative sputum bacilloscopy and that BAL performance should be a routine procedure as it is simple and usually uncomplicated technique.[15] Among various bronchoscopic specimens, BAL is considered best for diagnosis of TB.[7]

In one study, a BAL sample had significantly higher yield than bronchial wash. The higher yield is said to be due to large volume of saline used and less use of the anesthetic agent.[16] Bronchoscopic samples had a lower yield in several studies but at the same time some of studies had significant result and have emphasized the usefulness of BAL samples in the diagnosis of sputum smear negative pulmonary tuberculosis.

Wallace *et al*. as well as Kennedy *et al* and Vijayan *et al*,[14–18] have demonstrated lower yield whereas Baughman *et al*. reported 87% of bronchoscopy sample positivity in sputum smear negative cases.[19] A study by Mohan *et al*. confirmed PTB in 22 of the 50 patients from BAL, using the decision analysis approach, and suggested use of early BAL sample when the diagnosis of PTB is uncertain.[20] BAL had significant sensitivity and specificity in a study by Conde *et al*. and was useful in diagnosis of PTB in 72% cases.[21] In a study from Turkey, culture of BAL specimens was found to have sensitivity higher than induced sputum specimens.[22] In our study, the sensitivity was higher than the study by Mohan *et al*. and Conde *et al*. Small sample size could be the reason for it.

Fiberoptic bronchoscopy is useful in establishing accurate and early diagnosis of lower respiratory tract infections In our study, no complications occurred among patients undergoing bronchoscopy which is similar to a study by Anderson and coworkers[23] although minor side effects have been reported by Conde *et al*.

Most contraindications of bronchoscopy are relative and can be avoided with proper planning and preparation. All flexible bronchoscopes are high quality and perform well in the hands of an experienced brochoscopist; therefore bronchoscopy should be performed whenever the benefits of bronchoscopy outweigh the risk.
